# Transcriptome analysis of two cultivars of tobacco in response to *Cucumber mosaic virus* infection

**DOI:** 10.1038/s41598-019-39734-w

**Published:** 2019-02-28

**Authors:** Dan Liu, Qiang Zhao, Yazeng Cheng, Dandan Li, Caihong Jiang, Lirui Cheng, Yuanying Wang, Aiguo Yang

**Affiliations:** Tobacco Research Institute, Chinese Academy of Agricultural Sciences, Qingdao, 266101 China

## Abstract

*Cucumber mosaic virus* (CMV) is among the most important plant virus infections, inducing a variety of disease symptoms. However, the molecular mechanisms underlying plant responses to CMV infection remain poorly understood. In this study, we performed RNA sequencing analysis of tolerant (Taiyan8) and susceptible (NC82) tobacco cultivars on CMV-infected plants, using mock-inoculated plants as a control. The propagation of CMV in inoculated leaves did not show obvious difference between two cultivars, whereas virus accumulation in systemic leaves of Taiyan8 was smaller than those of NC82 at the same time point. We observed 765 and 1,011 differentially expressed genes (DEGs) in Taiyan8 and NC82, respectively, in CMV-inoculated leaves. DEGs related to reactive oxygen species, salicylic acid signal transduction, and plant–pathogen interaction were upregulated or downregulated in Taiyan8, which indicates that defense response pathways to CMV were activated in the tolerant cultivar. In addition, we identified several DEGs related to disease defense and stress resistance showing opposing expression patterns in the two cultivars. Our comparative transcriptome analysis will improve our understanding of the mechanisms of CMV tolerance in plants, and will be of great importance in the molecular breeding of CMV- tolerant genotypes.

## Introduction

Viruses can cause a variety of diseases in plants, and virus infection results in a range of symptoms^1^. Mosaic plant viruses can induce formation of discrete regions of dark green tissues in infected plants^2^. *Cucumber mosaic virus* (CMV) is a member of one of the most important virus families, Bromoviridae, which can infect at least 1,200 susceptible species in more than 100 plant families including crops, fruits, vegetables, ornamentals, woody plants, and other economically important plants worldwide^3–5^. CMV can cause severe systemic mosaic symptoms such as leaf distortion, systemic necrosis, chlorosis, dwarfism, and fruit lesions, thereby leading to drastic yield reduction^2,6^. CMV can be transmitted by at least 80 aphid species in a non-persistent manner^4^; it is also transmitted by the parasitic plant dodder (*Cuscuta* spp.) and in seeds^4^. Under experimental conditions, it can be established by mechanical inoculation using sap, purified virions, or viral RNA^2^.

CMV is among the best-characterized tripartite positive-sense single-stranded RNA viruses^4^. The CMV genome contains three segments: RNA1 (3.4 Kb), RNA2 (3.0 Kb), and RNA3 (2.2 Kb), and also includes two subgenomic RNAs: RNA4 (1.0 Kb) and RNA4A (0.7 Kb). RNA1, RNA2, and RNA3 can encode proteins 1a (111 kDa), 2a (97 kDa), and 3a (30 kDa), and proteins 2b (15 kDa) and 3b (25 kDa) are translated from subgenomic RNA4A and RNA4, respectively^2,4^. Proteins 1a and 2a are crucial for translation and synthesis of positive-strand RNAs^7^. The 2b protein is an RNA silencing suppressor that is involved in viral long-distance movement and inhibits the activity of small interfering RNA and Argonaute^8–12^. The 2b protein also suppresses the salicylic acid (SA) and jasmonic acid defense pathways in CMV-infected host plants^13,14^. Protein 3a is essential for viral intercellular movement^15^. Protein 3b, the capsid protein, is required for intercellular and long-distance movement and aphid transmission^2,16^. Of these, five proteins are important for the viral infection of different hosts. To survive, plants have evolved multiple sophisticated and complex regulatory mechanisms to defend against CMV infection including gene silencing pathways, hormone-mediated signaling pathways, and metabolism regulation^17,18^.

To date, studies of the molecular basis of CMV tolerance have focused mainly on qualitative resistance and some genes resistant to CMV infection have been isolated from *Arabidopsis thaliana* and common bean. For example, *RCY1*, which is an R gene containing the coiled coil-nucleotide binding site-leucine-rich repeat (LLR)-type protein in the C24 *A. thaliana* ecotype, mediates resistance against the yellow cucumber mosaic virus strain (CMV-Y)^19,20^. A TIR-NBS-LRR gene, *RT4–4*, is involved in CMV resistance response in *Phaseolus vulgaris*^21^. The *CUM1* and *CUM2* genes encode eIF4E and eIF4G, respectively, and The *cum1* and *cum2* mutations inhibit CMV multiplication in *Arabidopsis*^22^. Also in *Arabidopsis*, the transcription factor homeodomain-leucine zipper protein 1 plays a negative role in the anti-CMV defense response^18^, and the Pumilio protein APUM5 suppresses CMV infection via direct binding of viral RNAs^23^. However, to our knowledge, no such genes resistant to CMV have been found in tobacco germplasm. The *Nicotiana tabacum* cv. Taiyan8 is a putative CMV-tolerant tobacco variety. Although its tolerance to CMV appears to be a quantitative trait controlled by multiple genes^24^, the genetic control of tolerance is complex and remains largely unknown. Hence, it is necessary to attain a comprehensive understanding of the molecular mechanisms of tolerance in tobacco. Recently, RNA-sequencing (RNA-Seq) technology and digital gene expression analysis have provided new and rapid approaches for detecting differences in gene expression^25–27^. The global investigation of gene expression during CMV infection will help to elucidate the mechanisms of CMV tolerance in plants. Transcriptome analysis of *N*. *tabacum* infected by CMV during systemic symptom development has been reported^28^. However, a global comparison of gene expression in inoculated leaves of CMV-infected *N*. *tabacum* plants between susceptible and tolerant cultivars offered no clear results.

The tobacco cultivar Taiyan8, a main source of tolerance to CMV in tobacco breeding in China, exhibits moderate tolerance to CMV. The tolerance to CMV was inherited from Holmes, a tobacco line pyramided five CMV resistant loci^29^. In this study, we used next-generation deep sequencing approaches on two tobacco varieties, Taiyan8 and NC82 (a susceptible cultivar), to analyze responses to CMV infection at the transcriptome level. We investigated differences in gene expression between virus-infected samples and mock-inoculated samples at different time points following CMV infection (1, 3, and 5 days). Our results showed that some plant defense and disease resistance genes were differentially expressed between the two cultivars. Our study provided insight into the molecular mechanism of tobacco leaf response/resistance to CMV infection, and will further the current understanding of plant–virus interactions.

## Results

### Symptoms of inoculated tobacco leaves and virus detection

**Figure 1:**
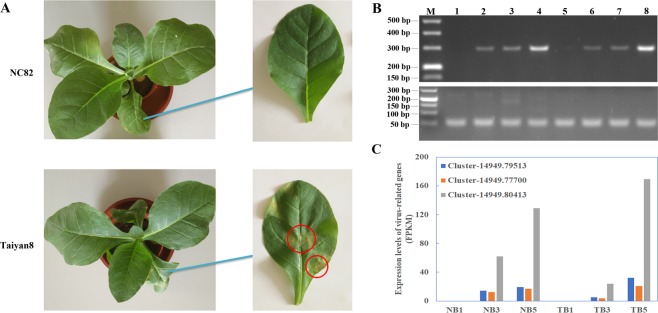
Symptoms of *Cucumber mosaic virus* (CMV) infection and accumulation of viral RNA in inoculated leaves of NC82 and Taiyan8. (**A**) Symptoms of CMV infection on inoculated leaves of two cultivars at 5 days post inoculation (dpi). Hypersensitive response like necrotic lesions on the inoculated Taiyan8 leaves at 5 dpi are indicated by the red cycles. (**B**) Semiquantitative RT-PCR detection results of inoculated leaves. The upper and lower lane indicates the amplification results with CMV *coat protein* gene and *NtEF1α* specific primers, respectively. M, DNA molecular weight marker; 1–4, mock-inoculated, CMV-inoculated at 1 dpi, CMV-inoculated at 3 dpi, and CMV-inoculated at 5 dpi of NC82 leaf; 5–8, mock-inoculated, CMV-inoculated at 1 dpi, CMV-inoculated at 3 dpi, and CMV-inoculated at 5 dpi of Taiyan8 leaf. The full-length gels are presented in Supplementary Fig. 2A,B. (**C**) Expression levels (FPKM) of CMV-associated genes in inoculated leaves of NC82 (NB) and Taiyan8 (TB) according to RNA-sequencing data. Cluster-14949.79513 is predicted to encode the CMV strain CTL segment RNA1; cluster-14949.77700 is predicted to encode the CMV isolate RP10 segment RNA2; cluster-14949.80413 is predicted to encode the *CMV 3a* gene for movement protein and the *cp* gene for coat protein.

After about 30 days post-inoculation (dpi), NC82 showed severe chlorosis and leaf distortion in systemic leaves, while Taiyan8 only showed slighter chlorosis and leaf vein clearing (Supplementary Fig. 1), which indicated that Taiyan8 showed a higher level of tolerance to CMV than NC82. When 40-day-old tobacco plants were mechanically inoculated with CMV, the inoculated leaves of NC82 showed no obvious symptoms at 5 dpi. However, tissues around the leaf vein showed hypersensitive response (HR) like necrotic lesions, which was similar to the response to CMV-Y in inoculated leaves of *Arabidopsis* ecotype C24 following viral infection^20^, was observed in inoculated leaves of Taiyan8 at 5 dpi (Fig. 1A).

To confirm whether CMV inoculation was successful, the presence of CMV virion in both virus- and mock-inoculated samples were confirmed by both semiquantitative reverse transcription PCR (RT-PCR) and read numbers associated with CMV from RNA-Seq data. The CMV inoculated leaves of two cultivars gained PCR products at 1, 3, and 5 dpi, while no virions were detected in the mock-inoculated leaves in either cultivar (Fig. 1B). The presence of CMV in virus-inoculated leaves of both cultivars at 3 and 5 dpi was also confirmed by the transcription data (Fig. 1C), in which three strands of CMV were detected in the assembled transcript dataset. The reads from CMV were absent from virus-inoculated leaves of both cultivars at 1 dpi, which might be due to the low virion content at the initial infection stage. These results indicated that the samples used for transcriptome analysis had been successfully inoculated. The presence of CMV in upper leaves were also detected using semiquantitative RT-PCR. The expression level of *cp* gene in upper leaf of NC82 was much higher than that of Taiyan8 (Supplementary Fig. 2C,D), which indicated that the contents of CMV in systemically infected leaves of Taiyan8 were lower.

### *De novo* assembly of the tobacco leaf transcriptome and functional annotation

To determine the molecular mechanisms underlying pathogenesis, inoculated leaves were harvested at 1, 3, and 5 dpi for RNA extraction from CMV-infected and mock-inoculated plants. To reduce biological errors caused by natural variation, three CMV-inoculated samples that exhibited similar symptoms and three mock-inoculation samples were collected and sequenced using the Illumina HiSeq™ 2000 sequencing system. Sequencing libraries were generated for cultivars Taiyan8 and NC82 from the total RNA of control and infected plants at 1, 3, and 5 dpi. About 1.32 and 1.34 billion raw reads for Taiyan8 and NC82, respectively, were generated from all sample cDNA libraries (Table 1). Then reads with adaptors, and unknown and low-quality bases were filtered out, leaving 1.31 and 1.33 billion clean reads for Taiyan8 and NC82, respectively. Clean data were submitted to the NCBI Sequence Reads Archive database (SRP126702). In addition, 48 sequencing libraries were generated for symptomatic leaves collected at 1, 3, 8, and 15 dpi. These libraries provided another set of clean data for submission to NCBI (SRP126464). All clean reads from the 84 sequencing libraries were used to assemble the transcriptome data using the Trinity program. Using overlapping information in high-quality reads, 377,547 transcripts and 359,112 assembled unigenes were obtained (Supplementary Table 1). The assembled transcriptome sequences were used as reference sequences. The clean data were mapped back to the assembled transcriptome. At least 79.05% of the data were mapped back to the assembled transcriptome (Supplementary Table 2). These results demonstrated the high quality of the transcriptome assembly. To annotate the assembly unigenes, BLAST results were analyzed against seven databases: Nr (NCBI non-redundant protein sequences database), Nt (NCBI nucleotide sequences database), Pfam (protein family database), Swiss-Prot (A manually annotated and reviewed protein sequence database), KOG (euKaryotic Ortholog Groups), GO (Gene Ontology), and KO (Kyoto Encyclopedia of Genes and Genomes [KEGG] Ortholog database). The results showed that 267,674 (74.53%) unigenes matched one or more of the databases; a total of 25,730 (7.6%) unigenes were annotated in all seven databases (Supplementary Fig. 3).

**Table 1:** Statistics describing Illumina sequencing data.

Sample	Raw reads	Clean reads	Clean bases	Q20 (%)	Q30 (%)	GC content (%)	Notes
NBC1–1	80290422	78411710	7.84G	98.55	96.16	43.7	Replicate 1
NBC1–2	74093220	71557292	7.16G	98.58	96.24	43.4	Replicate 2
NBC1–3	66827226	65276484	6.53G	98.49	96.06	43.9	Replicate 3
NBC3–1	68435298	67298720	6.73G	98.14	95.23	43.6	Replicate 1
NBC3–2	68464894	67324834	6.73G	98.07	95.05	43.3	Replicate 2
NBC3–3	61752242	60692282	6.07G	97.44	93.53	43.9	Replicate 3
NBC5–1	89885612	87643800	8.76G	98.85	96.93	43.9	Replicate 1
NBC5–2	76934672	75387696	7.54G	98.84	96.90	43.2	Replicate 2
NBC5–3	81765444	80225336	8.02G	98.92	97.09	43.0	Replicate 3
NB1–1	84625592	83146050	8.31G	98.39	95.81	44.2	Replicate 1
NB1–2	69842568	69294324	6.93G	98.37	95.81	44.2	Replicate 2
NB1–3	69880060	68128674	6.81G	98.52	96.14	44.0	Replicate 3
NB3–1	64342448	63304072	6.33G	98.31	95.56	43.9	Replicate 1
NB3–2	76916300	74071664	7.41G	98.33	95.60	43.2	Replicate 2
NB3–3	84759674	83339370	8.33G	98.35	95.67	43.4	Replicate 3
NB5–1	61243574	60357490	6.04G	98.02	94.99	43.3	Replicate 1
NB5–2	81789376	80186954	8.02G	98.89	96.98	43.4	Replicate 2
NB5–3	74426836	73091330	7.31G	98.79	96.73	43.6	Replicate 3
TBC1–1	91716968	86830442	8.68G	98.55	96.13	43.5	Replicate 1
TBC1–2	77969928	61100982	6.11G	96.77	92.54	44.4	Replicate 2
TBC1–3	76361004	74625470	7.46G	98.34	95.82	44.0	Replicate 3
TBC3–1	74404112	71612746	7.16G	98.31	95.51	43.9	Replicate 1
TBC3–2	65825154	64511680	6.45G	97.73	94.20	43.6	Replicate 2
TBC3–3	78966092	77651532	7.77G	98.43	95.79	43.9	Replicate 3
TBC5–1	75155008	73584628	7.36G	98.09	95.08	43.6	Replicate 1
TBC5–2	73057930	71941468	7.19G	98.08	95.08	43.5	Replicate 2
TBC5–3	71677160	71396726	7.14G	97.98	94.89	43.7	Replicate 3
TB1–1	63917904	62389162	6.24G	98.42	95.92	44.0	Replicate 1
TB1–2	71621450	70452578	7.05G	98.65	96.42	44.0	Replicate 2
TB1–3	75924438	74267020	7.43G	96.76	92.74	43.8	Replicate 3
TB3–1	64732692	63374188	6.34G	98.26	95.46	43.5	Replicate 1
TB3–2	78543212	76620158	7.66G	98.45	95.85	44.0	Replicate 2
TB3–3	68891160	67429888	6.74G	97.88	94.44	43.7	Replicate 3
TB5–1	77607976	76454506	7.65G	97.88	94.70	43.8	Replicate 1
TB5–2	65087654	63879292	6.39G	97.99	94.79	43.3	Replicate 2
TB5–3	68514354	67585204	6.76G	98.06	95.05	43.5	Replicate 3

NBC and TBC represent NC82 and Taiyan8 mock-inoculated leaves, respectively; NB and TB represent NC82 and Taiyan8 leaves infected with *Cucumber mosaic virus* (CMV), respectively; The number 1, 3, or 5 represent mock- or CMV-inoculated leaves at 1, 3, or 5 days post inoculation, respectively.

### Differentially expressed genes for CMV infection at different time points

**Figure 2:**
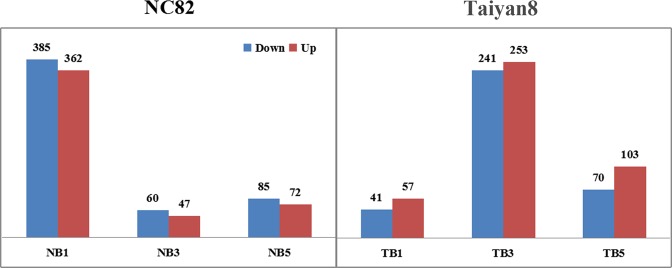
Differentially expressed genes in Taiyan8 and NC82. NB1, NB3, and NB5 represent comparisons between NC82 Cucumber mosaic virus (CMV)-inoculated leaves and mock-inoculated leaves at 1, 3, and 5 dpi, respectively; the same comparisons were performed between CMV- and mock-inoculated Taiyan8 leaves at 1, 3, and 5 dpi (TB1, TB3, and TB5, respectively).

**Figure 3:**
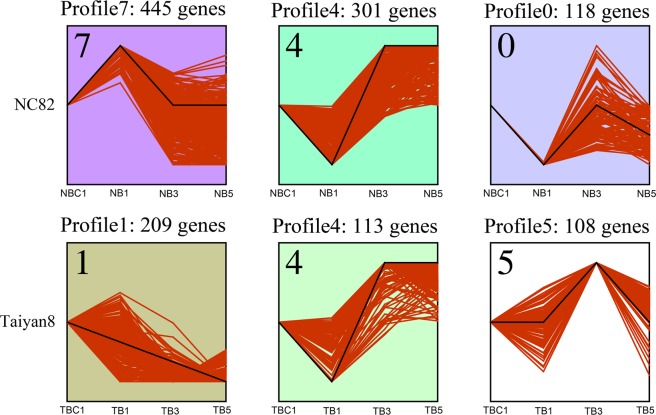
Patterns of gene expressions across time points in NC82 and Taiyan8 inferred by STEM analysis (*p* < 0.01). The black line represented the expression tendency of all the genes. The red line represented each individual gene that was scaled separately to be closely aligned with the model profile. The number of genes belonging to each pattern was labeled above the frame.

To eliminate the effects of genetic differences and development stage, differentially expressed genes (DEGs) were identified by comparing CMV-inoculated and mock-inoculated cultivars at the same time points. A total of 1,776 DEGs were obtained between CMV-infected and mock-inoculation samples (Fig. 2, Supplementary Tables 3 and 4). The number of downregulated DEGs in NC82 inoculated with CMV decreased from 385 at 1 dpi, to 60 at 3 dpi, and finally to 85 at 5 dpi. There were fewer upregulated DEGs than downregulated DEGs at 1 dpi (362 vs. 385 genes), 3 dpi (47 vs. 60 genes) and 5 dpi (72 vs. 85 genes) (Fig. 2). Taiyan8 inoculated with CMV contained a total of 98, 494, and 173 DEGs at 1, 3, and 5 dpi, respectively. Among these, 41 DEGs were downregulated and 57 upregulated at 1 dpi. A total of 253 DEGs were upregulated and 241 DEGs downregulated at 3 dpi; 103 DEGs were upregulated and 70 DEGs downregulated at 5 dpi (Fig. 2). The DEGs of NC82 and Taiyan8 from different stages were clustered into ten profiles based on gene expression patterns using STEM software. The profiles showed different patterns in gene expression over time in response to CMV between the two cultivars. Most of the DEGs were significantly overrepresented in the profile exhibiting apparent increase in expression levels at 1 dpi in NC82 (Profile7, *p* < 0.01), while in Taiyan8 most of the DEGs were significantly overrepresented in Profile1 (*p* < 0.01), in which gene expression levels decreased over the time course of infection (Fig. 3). In both cultivars, a large percentage of the DEGs were overrepresented in Profile4 (*p* < 0.01), in which the expression was suppressed at 1 dpi but induced at 3 dpi (Fig. 3).

**Figure 4:**
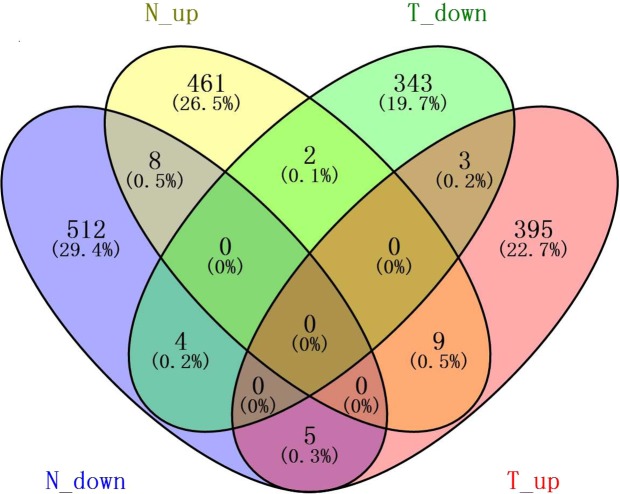
Venn diagram showing commonalities and differences among four lists of shared differentially expressed genes (DEGs). N_up, genes upregulated in NC82 at each time point; N_down, genes downregulated in NC82 at each time point; T_up, genes upregulated in Taiyan8 at each time point; T_down, genes downregulated in Taiyan8 at each time point. The numbers in the brackets represents the ratio of related genes to total DEGs.

In total, 529 and 352 DEGs were downregulated and 480 and 412 DEGs were upregulated in NC82 and Taiyan8, respectively, regardless of time point. The shared DEGs were further analyzed to examine their commonalities and differences. As shown in the Venn diagram in Fig. 4, nine genes were upregulated and four genes were downregulated in both NC82 and Taiyan8. Seven genes mainly associated with disease defense and stress resistance showed opposing expression patterns in the two cultivars. The gene Cluster-14949.112502, which was predicted to encode glycosyltransferase, was induced in NC82 but suppressed in Taiyan8. Glycosyltransferases play a major role in buffering the impacts of biotic and abiotic stresses on plants through the glycosylation of small molecules including secondary metabolites and hormones^30^. Four genes (Cluster-14949.87764, Cluster-14949.271942, Cluster-14949.227738, and Cluster-14949.40413), which encode the elicitor-responsive protein (ERG), ammonium transporter (AMT), β-1,3-glucanase, and peroxisomal (S)-2-hydroxy-acid oxidase, respectively, were upregulated in Taiyan8 but downregulated in NC82 (Supplementary Table 5). In rice, ERG1 protein is involved in plant defense signaling systems^31^. AMT proteins are involved in a diversity of aspects of plant growth and development. In *Arabidopsis*, the AMT1.1 protein is involved in *Pseudomonas syringae*- and *P*. *cucumerina*-mediated disease^32,33^. Tobacco β-1,3-glucanase is coordinately increased in response to ethylene and SA, which is implicated in the defense response against pathogens^34^. Peroxisomal (S)-2-hydroxy-acid oxidase is involved in peroxisome metabolism and may modulate H_2_O_2_ levels in rice in association with catalase^35^.

### GO and KEGG enrichment analysis of DEGs

**Figure 5:**
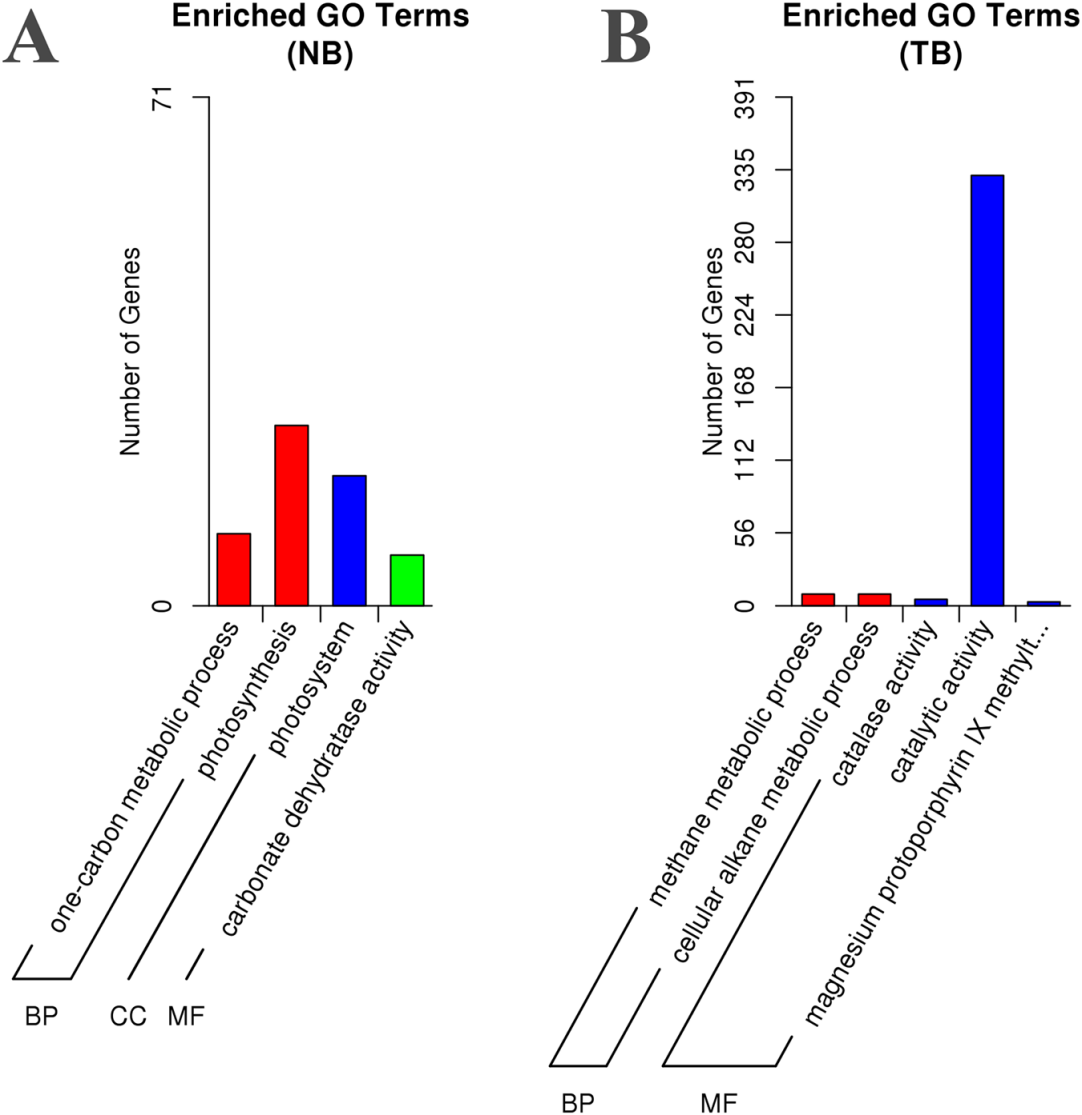
Gene ontology (GO) enrichment of differentially expressed genes of Taiyan8 and NC82. (**A**) Enriched GO terms in NC82. (**B**) Enriched GO terms in Taiyan8.

GO and KEGG enrichment analyses were performed to determine the biological functions of the identified DEGs. In total, 707 and 559 DEGs were annotated with at least one GO term in NC82 and Taiyan8, respectively. In NC82, only four GO terms were significantly enriched including a one-carbon metabolic process and photosynthesis as biological processes, carbonate dehydratase activity as a molecular function, and photosystem as a cellular component (Fig. 5A). In Taiyan8, prominent biological processes included a methane metabolic process and cellular alkane metabolic process (Fig. 5B). Among molecular functions, the majority of DEGs were enriched in catalytic activity (GO:0003824). Catalase activity and magnesium protoporphyrin IX methyltransferase activity were also significantly enriched (Fig. 5B, Supplementary Table 6) after CMV inoculation, which indicates that genes in these processes may play pivotal roles in response to CMV infection. KEGG pathway analysis showed that 10 pathways among the top 20 metabolism pathways with the highest number of DEGs were common between the cultivars (Supplementary Table 7). The common pathways were related to signal transduction (ko04075), carbohydrate metabolism (ko00630, ko00620), secondary metabolite biosynthesis (ko00940), nucleotide metabolism (ko00230, ko00240), porphyrin and chlorophyll metabolism (ko00860), genetic information processing (ko03013, ko04141), and cellular processing (ko04146). The remaining DEGs in NC82 were mainly related to energy metabolism (ko00195, ko00196, and ko00910), amino acid metabolism (ko00480, ko00270, and ko00280), carbohydrate metabolism (ko00010, ko00051), the mRNA surveillance pathway (ko3015), and lipid metabolism (ko00564). The most enriched pathways of the remaining Taiyan8 DEGs were mainly located in genetic information processing (ko03008, ko03040, ko03010), cellular processes (ko04144), carbohydrate metabolism (ko00500), energy metabolism (ko00710, ko00190), and plant-pathogen interaction (ko04626) (Supplementary Table 7). In summary, based on GO and KEGG pathway analyses, the difference in response to CMV between NC82 and Taiyan8 mainly involved photosynthesis, reactive oxygen species (ROS) scavenging, plant hormone signal transduction, and plant–pathogen interaction.

### Identification of DEGs involved in photosynthesis

Based on GO and KEGG pathway analyses, photosynthesis was differentially regulated in NC82 and Taiyan8 after CMV infection. About 60 DEGs were identified as related to photosynthesis in the two cultivars including genes coding antenna proteins, photosystem II-related proteins, chlorophyll biosynthesis proteins, and carbon assimilation proteins. The DEGs associated with photosynthetic antenna proteins and photosystem II were upregulated at 1 dpi in NC82, but expression was not significantly changed in Taiyan8 at the same time point (Supplementary Table 8). In NC82 at 1 dpi, the increased expression of genes involved in photosynthesis was coupled with increased expression of those involved in chlorophyll synthesis and carbon assimilation (magnesium chelatase, uroporphyrinogen decarboxylase, triosephosphate isomerase, fructose-bisphosphate aldolase). In fact, most of the genes related to chlorophyll biosynthesis and carbon fixation (Cluster-14949.123042, Cluster-14949.194004, Cluster-14949.195585, Cluster-14949.186780, Cluster-14949.186620, and Cluster-14949.179959) remained unchanged at 1 dpi and were downregulated at 3 dpi in Taiyan8.

### Identification of DEGs involved in ROS scavenging

ROS are important signaling molecules that regulate the onset of HR cell death^36^. To cope with ROS toxicity, plants have evolved a sophisticated system involving enzymatic and non-enzymatic antioxidants. In Taiyan8, GO annotation indicated that catalase activity was significantly enriched, whereas more DEGs associated with glutathione metabolism (ko00480) were enriched in NC82. In total, five DEGs predicted to encode catalase were identified in Taiyan8, whereas only one DEG-encoding catalase was identified in NC82. Noticeably, the expression levels of all identified CATs were suppressed at 1 or 3 dpi. Glutathione and ascorbate play important roles as non-enzymatic antioxidants in ROS scavenging. In NC82, 11 DEGs were related to glutathione and ascorbate, most of which were downregulated at 1 dpi including glutathione transferase (GST), ascorbate peroxidase, and glutathione reductase. In Taiyan8, two DEGs were found to encode GST (one was downregulated at 5 dpi, one was upregulated at 5 dpi) and two DEGs (downregulated at 3 dpi) encoded monodehydroascorbate reductases (Supplementary Table 8).

### Identification of DEGs involved in plant hormone signal transduction

Plant hormones play a pivotal role in plant–pathogen interactions^37^. Our transcriptome analysis indicated that twice as many DEGs were involved in plant hormone signal transduction in NC82 than in Taiyan8 (Supplementary Table 8). In NC82, genes encoding auxin-induced proteins, auxin-responsive proteins, auxin response factor, abscisic acid insensitive protein, and receptors of gibberellin, cytokinin, and ethylene exhibited significantly different expression in response to CMV infection. Interestingly, one non-expressor of a pathogenesis-related gene (NPR3) was inhibited at 3 dpi in Taiyan8 but remained unchanged in NC82.

### Identification of DEGs involved in plant-pathogen interaction

In this study, we identified five and two DEGs in the plant–pathogen interaction pathway (ko04626) in Taiyan8 and NC82, respectively (Supplementary Tables 7 and 8). In both cultivars, DEGs encoding cyclic nucleotide-gated channel proteins were identified; both were upregulated. In Taiyan8, two DEGs (Cluster-14949.184690 and Cluster-14949.205754) encoding respiratory burst oxidase, which might be involved in HR reactions, were induced at 3 dpi. We also found two DEGs that encoded the disease resistance RPM1-interacting protein (Cluster-14949.119142) and pathogenesis-related gene transcriptional activator PTI5 (Cluster-14949.287620) in Taiyan8. In NC82, one gene encoding LRR receptor-like kinase was upregulated at 5 dpi.

### Validation of DEGs by quantitative PCR

**Figure 6:**
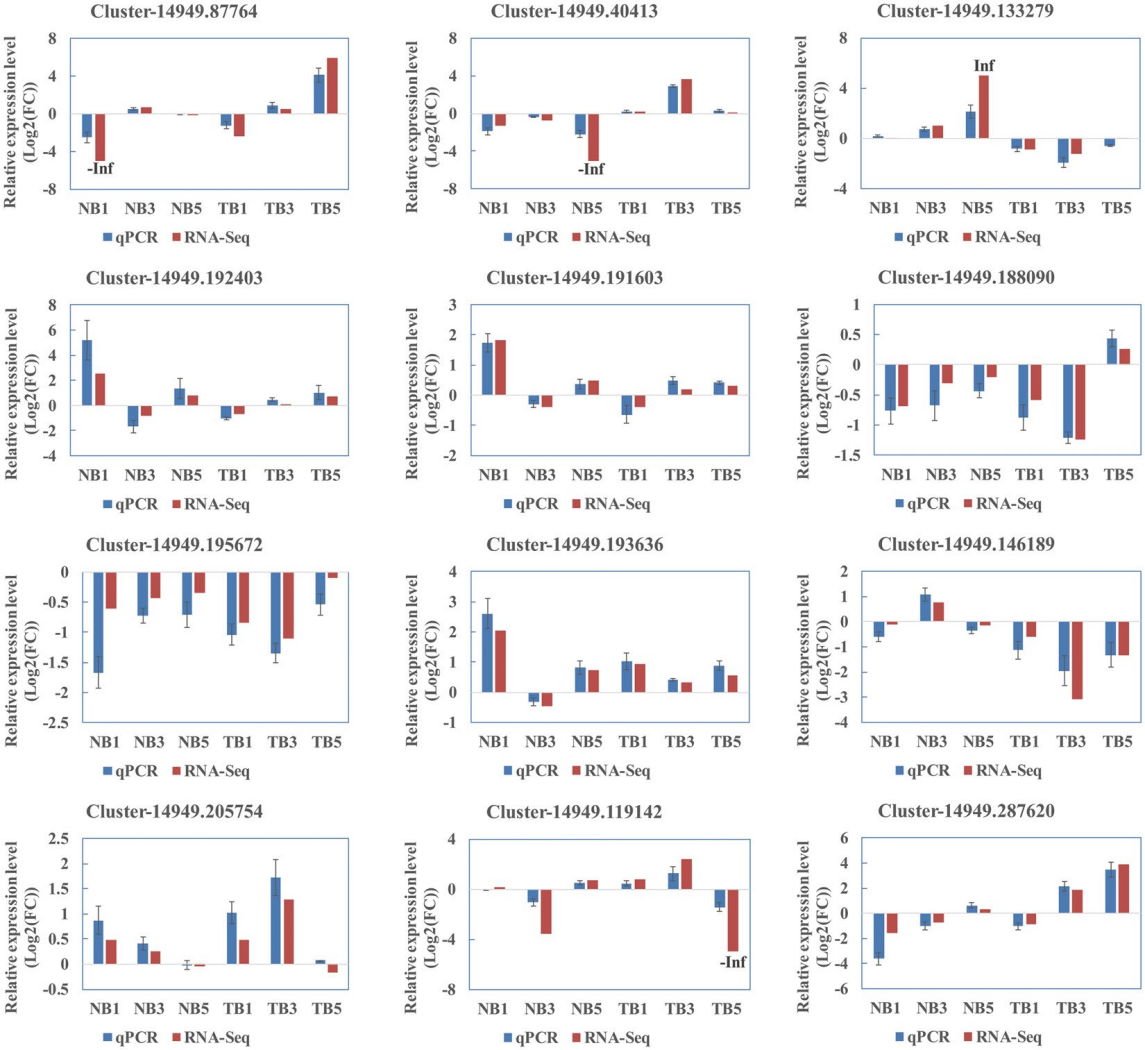
Validation of the expression of selected genes from RNA-sequencing (Red) using real-time quantitative reverse transcription (qRT)-PCR (Blue). Error bars represent the standard errors of the qRT-PCR signals. The *NtEF1α* gene was used as a control to normalize the qRT-PCR analysis.

RNA-Seq revealed the expression profiles of thousands of genes. To validate the DEG result, 12 genes involved in photosynthesis, reactive oxygen scavenging, signal transduction, and plant-pathogen interaction were selected for real-time quantitative reverse transcription PCR (qRT-PCR) analyses using specific primers (Supplementary Table 9). The results of both RNA-Seq and qRT-PCR showed that all the genes were differentially expressed with a concordant direction of fold change (Fig. 6), which indicates that the RNA-Seq results were reliable.

## Discussion

CMV, as a systemically infectious virus, causes economic losses worldwide^3^. To identify genes involved in broad-spectrum tolerance to CMV, three previous studies investigated tobacco plant responses to CMV and the mechanisms underlying disease development using next-generation sequencing to monitor transcriptional changes in systemically infected leaves, with the results that thousands of DEGs were discovered^28,38,39^. In this study, we used RNA-Seq approaches to analyze gene expression changes in CMV-inoculated leaves between two cultivars. The results will assist in the discovery of important genes in plant defense response and the elucidation of the underlying mechanisms of CMV infection responses in inoculated leaves.

After CMV infection, the symptoms in systemically infected leaves of Taiyan8 developed later and less severity (Supplementary Fig. 1). The virus accumulation in the upper leaves of Taiyan8 was smaller than those of NC82 at the same time point (Supplementary Fig. 2C,D), whereas the propagation of CMV in inoculated leaves of Taiyan8 was not inhibited compared to that of NC82 (Fig. 1B,C). These results suggested that the systemic spread of CMV or the propagation of virus in upper leaves in Taiyan8 was suppressed, which may contribute to the less severe symptoms.

In Taiyan8, we observed necrotic lesions in inoculated leaves at 5 days after CMV infection, whereas the susceptible cultivar, NC82, showed no obvious symptoms in CMV-inoculated leaves (Fig. 1). The necrosis might have been induced by HR or was merely a symptomatic response to CMV infection. HR is a form of programmed cell death that occurs during a plant’s resistance response to pathogens^40^. ROS are important signaling molecules that regulate the onset of HR cell death^36^. NADPH oxidases (i.e., respiratory burst oxidase homologues [RBOHs]) are the most important enzymatic ROS-generating systems in plants^41^. In *Arabidopsis* ecotype C24, NADPH oxidase was significantly induced in *RCY1*-mediated CMV resistance by forming HR-induced necrotic spots in inoculated leaves following viral inoculation^42^. In this study, two DEGs encoding RBOHs involved in the plant–pathogen interaction pathway (ko04626) were upregulated at 3 dpi in Taiyan8. Necrosis induced by HR often confined the spread of the virus and enhanced resistance in other regions of the plant^42^. In *Arabidopsis*, however, *HRT* mediates HR after turnip crinkle virus (TCV) infection and is necessary but generally insufficient for resistance to TCV. Plants containing only *HRT* developed HR but were susceptible to the virus^43,44^. In addition, five and one DEGs predicted to encode catalase were identified in Taiyan8 and NC82, respectively, and their expression levels were all downregulated. Catalase (CAT) is the main enzyme that breaks down H_2_O_2_. The induced expression levels of *RBOH*s and downregulation of CAT could further enhance ROS accumulation, which would trigger a localized oxidative burst and cell death during HR^45^. In comparison, 2b proteins of some CMV strains (e.g., CMV-HL) interact with CAT3 in *Arabidopsis*^46,47^. Direct interaction between 2b proteins and CAT3 induced HR-like necrosis on both inoculated and upper leaves. The 2b-mediated necrosis was thought to be a detriment, rather than a potential defense mechanism, as it did not restrict CMV at all in *Arabidopsis*^46^. Whether 2b proteins of the CMV strain used in this investigation interacted with CAT in Taiyan8 remains unclear. However, note that the CMV-HL strain also induced distinct necrosis on upper leaves of *Arabidopsis*^46^, whereas in our study, upper leaves of Taiyan8 exhibited chlorosis but not necrosis, which suggests that the pathways of necrotic lesion formation in tobacco might be different from those of *Arabidopsis*. Noticeably, the symptoms on upper leaves were less severe in Taiyna8 than those in NC82 at the later infection stages (Supplementary Fig. 1), which indicates that tolerance to CMV in Taiyan8 might be partially due to the formation of necrotic lesions in inoculated leaves.

CMV infection disturbed plant hormone signal transduction in both cultivars. In the susceptible cultivar, several DEGs related to auxin transport and response were found to have been induced after CMV infection. Auxins are involved in symptom development during virus infection^48^. Viruses manipulate functions and subcellular localization of certain auxin factors to promote their own replication and dissemination^37^. Viral manipulation of auxin response factors accounts for the symptoms observed after viral infection^37^. The induced expression levels of auxin-related genes in NC82 could enhance symptom severity. *NPR3*, a key receptor of SA, negatively regulates *PR* gene expression and pathogen resistance in *Arabidopsis* by interacting with TGA2 and its paralogs^49,50^. The knockout of *NPR3/NPR4* leads to elevated *PR* expression and enhances resistance against pathogens^50^. It has been also demonstrated that an essential function of *TGA2* is the positive regulation of systemic acquired resistance and negative regulation of the basal expression of *PR1*^51^. In this study, one *NPR3* (Cluster-14949.146189) and one *TGA2* (Cluster-14949.236418) were downregulated in Taiyan8 (Supplementary Table 8). The downregulation of these two genes may elevate the basal *PR* gene expression levels. To support these results, we found that one gene encoding β-1,3-glucanase (PR2) was upregulated in Taiyan8 at 5 dpi (Supplementary Table 5). We also identified one gene encoding pathogenesis-related gene transcriptional activator Pti5 that was weakly upregulated at 3 dpi and significantly upregulated at 5 dpi in Taiyan8. The Pti5 proteins belong to the ethylene-response factor family and positively regulate the expression of *PR1* and *PR2*^52^. These results indicate that NPR3–TGA2 interaction may contribute to CMV disease tolerance in the tolerant cultivar.

Photosynthesis was one of the most significantly responsive processes in terms of gene expression level following CMV inoculation in NC82 (Supplementary Tables 6 and 7). The downregulation of photosynthesis-related genes has been observed regularly in chlorotic leaves of tobacco following CMV attack^28,53^. We observed a large increase in photosynthesis-related gene expression levels in inoculated leaves of susceptible tobacco cultivars at 1 day after virus inoculation (Supplementary Table 8). Gene expression levels were downregulated at 3 dpi compared to at 1 dpi in NC82 (data not shown), whereas in Taiyan8, the genes involved in photosynthesis did not exhibit a significant change in inoculated leaves. Similarly, PVY-inoculated potato leaves showed a transient increase in photosynthesis-related gene expression immediately after virus infection^54^. Upregulation during the early stage of infection in NC82 may have been the consequence of a stress response triggering an increase in energy consumption^54^.

## Conclusions

In this study, we comparatively analyzed gene expression profiles of inoculated leaves of CMV-susceptible and -tolerant tobacco cultivars during different stages of CMV infection. Our results showed that 765 and 1,011 DEGs were identified upon CMV infection in Taiyan8 and NC82, respectively. There were more DEGs in NC82 than in Taiyan8 at 1 dpi, which implied that the CMV-tolerant cultivar was less affected by CMV infection in the initial stage of inoculation. Functional annotation analysis showed that DEGs related to ROS, plant hormone signal transduction, and plant–pathogen interactions showed different expression patterns in the two cultivars, which may be involved in the defense response pathways to CMV in the tolerant cultivar. We also identified several DEGs related to disease defense and stress resistance, which showed opposing expression patterns in the two cultivars. Our genome-wide transcriptome analysis will assist in the discovery and annotation of important plant disease response genes and provide a scientific basis for further investigation of the molecular mechanisms underlying CMV infection in tobacco. Further studies should focus on whether and how these pathogen-related genes play essential roles in the interaction between viruses and host plants.

## Materials and Methods

### Plant materials and virus inoculation

We used *N*. *tabacum* L. cv. NC82 (susceptible to CMV) and *N*. *tabacum* L. cv. Taiyan8 (tolerant to CMV) in the experiments. The CMV virus source belonged to subgroup IB was purchased from Chinese Academy of Inspection and Quarantine and preserved on *N*. *tabacum* L. cv. Samsun NN. Tobacco seeds were surface-sterilized with 3.0% NaClO for 5–10 min, and subsequently sown onto plates containing Murashige and Skoog media containing 3% sucrose and 0.6% agar under a controlled 16 h light/8 h dark cycle at 25 °C^55^. Then the seedlings were transferred to soil and grown in a chamber under 70–100 μM m^−2^ s^−1^ light intensity with 14 h of light at 26 °C and 10 h of darkness at 20 °C. After approximately 40 days, leaves from the bottom insertions were mechanically inoculated with CMV or mock-inoculated with phosphate buffer, as described previously^56^. Inoculated tobacco leaves were obtained at three time points: 1, 3, and 5 dpi. In total, 80 and 100 tobacco plants were CMV-inoculated and mock-inoculated, respectively. The sample leaves were immediately frozen in liquid nitrogen and stored at −80 °C until RNA extraction for RNA-Seq analysis. To verify successful inoculation, CMV- and mock-inoculated leaf samples and the upmost fully expanded leaf samples were detected via semiquantitative RT-PCR using CMV coat protein (CP) specific primers (forward primer: 5′-TACCCTGAAACCACCGAAAA-3′; reversed primer: 5′-CGCCGAAAGATCATACAACA-3′). The relative expression levels of the *ELONGATION FACTOR1α* gene (*NtEF1α*, GenBank Accession No. NM001326165) were determined using primers *EF1α*F and *EF1α*R and used as internal controls for the assay (Supplementary Table S9).

### RNA extraction, library construction, and Illumina sequencing

Frozen leaf samples were crushed and used for RNA extraction. Total RNA was extracted using TRIzol reagent (Invitrogen, Carlsbad, CA, USA) following the manufacturer’s protocol. RNA purification was performed using an RNeasy Mini Kit (QIAGEN, Chatsworth, CA, USA). RNA concentration was measured using the Qubit RNA Assay Kit and Qubit 2.0 Fluorometer (Life Technologies, Carlsbad, CA, USA). The RNA Nano 6000 Assay Kit (Agilent Technologies, Santa Clara, CA, USA) was used in the Agilent Bioanalyzer 2100 system (Agilent Technologies) to measure RNA integrity. For RNA-Seq analysis, mRNA was purified using poly T oligo-attached magnetic beads, and subsequently fragmented randomly into short pieces by adding fragmentation buffer. First-strand cDNA was synthesized using M-MuLV reverse transcriptase and random hexamer primers. Then DNA polymerase I, dNTPs, and RNase H were used to synthesize second-strand cDNA. After purification and end repair, the cDNA fragments were ligated to sequencing adapters. Then fragments of a suitable size (150–200 bp) were purified and amplified by PCR to obtain the final library. The quality of the library was tested on the Agilent Bioanalyzer 2100 system and clustering of the index-coded sample was performed using the cBot Cluster Generation System with the TruSeq PE Cluster Kit v3-cBot-HS (Illumina, San Diego, CA, USA) according to the manufacturer’s instructions. Finally, the library was sequenced using the HiSeq™ 2000 platform (Illumina) and 100 bp paired-end reads were generated.

### *De novo* transcriptome assembly and gene annotation

Raw data (raw reads) in fastq format were first processed through in-house Perl scripts. The raw reads containing adapter sequences, reads containing poly N, and then low-quality reads were removed to obtain clean reads. High-quality clean data were obtained by the calculation of Q20 and Q30 scores, GC-content, and sequence duplication level, and subsequently used in all downstream analyses. *De novo* transcriptome assembly was accomplished using Trinity^57^ with min_kmer_cov set to 2 by default and all other parameters in the default settings. Then the assembled contigs were hierarchically clustered by Corset^58^ using shared reads and expression data. The longest transcripts in the cluster units were considered unigenes to eliminate redundant sequences, and then were combined to produce the final assembly used for annotation. For gene annotation, gene functions were searched against the following databases: the NR NCBI protein database, NT NCBI database, PFAM, Swiss-Prot database, KO database, KOG NCBI database, GO database, and KEGG database.

### Differential expression, GO, and KEGG enrichment analysis

The DEG analysis between inoculated and mock-inoculated plants was performed using the DESeq^59^ package with the rigorous algorithm method. The *P*-value threshold was determined using Benjamini and Hochberg’s approach for controlling the false discovery rate (FDR). Significant DEGs were assigned using the following criteria: FDR < 0.05 and |log_2_(fold change)| ≥ 1. Gene expression patterns across time points in the two cultivars were analyzed by STEM^60^. Venn diagrams were drawn using the VENNY software (http://bioinfogp.cnb.csic.es/tools/venny). GO enrichment analysis, which was implemented using the *goseq* R package, based on the Wallenius non-central hypergeometric distribution, was used to annotate the DEGs at three levels (biological process, molecular function, and cellular component). *P*-values ≤ 0.05 were considered significantly enriched. For metabolic pathway analysis, all DEGs were determined using the KEGG database (http://www.genome.jp/kegg) and the statistical enrichment of DEGs was tested using the KOBAS software^61^.

### Gene expression qRT-PCR analysis

To validate the RNA-Seq results, we performed qRT-PCR analysis with the RNA samples that were used to prepare the sequencing libraries. First-strand cDNA was synthesized using the PrimeScript 1st Strand cDNA Synthesis Kit (TaKaRa, Dalian, China) according to the manufacturer’s instructions. The 20 µL qRT-PCR solutions contained SYBR Premix Ex Taq II (Tli RNaseH Plus) (2×) (TaKaRa), 0.8 μL forward and 0.8 μL reverse primers, 0.4 μL ROX Reference Dye II (50×) and 30 ng of cDNA template. qRT-PCR reactions (95 °C, 3 min; 95 °C, 5 s; 60 °C, 34 s; 40 cycles) were performed using the SYBR Green method on the Applied Biosystems 7500/7500 Fast Real-Time PCR System (Life Technologies). Relative gene expression analyses were calculated via the full quantification method using *NtEF1α*as the internal control gene. At least three biological replicates were performed for each individual experiment. Primers used for qRT-PCR are shown in Supplementary Table 9. The 2^−ΔΔCT^ method was used for relative quantification^62^.

## Supplementary information

Supplementary information

Supplementary Table 1

Supplementary Table 2

Supplementary Table 3

Supplementary Table 4

Supplementary Table 5

Supplementary Table 6

Supplementary Table 7

Supplementary Table 8

Supplementary Table 9

## Data Availability

The RNA-Seq raw data were deposited in the NCBI Sequence Read Archive (SRA) with the accession number SRP126464 and SRP126702.
