# Supplementary material for: Transcriptome analysis of two cultivars of tobacco in response to Cucumber mosaic virus infection
Source: Sci Rep. 2019 Feb 28;9:3124. doi: 10.1038/s41598-019-39734-w (PMC6395745; doi:10.1038/s41598-019-39734-w)
Supplement: Supplementary file 1 — Supplementary information [file 41598_2019_39734_MOESM1_ESM.docx]

***Supplementary Information***

**Transcriptome analysis of two cultivars of tobacco in response to *Cucumber mosaic virus* infection**

Dan Liu^†^, Qiang Zhao^†^, Yazeng Cheng, Dandan Li, Caihong Jiang, Lirui Cheng, Yuanying Wang, Aiguo Yang*****

**^*^Correspondence:** Aiguo Yang: yangaiguo@caas.cn

# Supplementary files

**Supplementary Figure 1**. Symptoms of *Cucumber mosaic virus* (CMV) infection on systemic leaves of NC82and Taiyan8 at 30 days post inoculation (dpi). NC82 showed severe chlorosis and leaf distortion; Taiyan8 showed slighter chlorosis and leaf vein clearing.

**Supplementary Figure 2**. The full-length agarose gel electrophoresis of semiquantitative RT-PCR detection results of inoculated and upmost fully expanded leaves. (A) The amplification results of inoculated leaf with CMV *coat protein* gene specific primers (F: 5′-TACCCTGAAACCACCGAAAA-3′; R: 5′-CGCCGAAAGATCATACAACA-3′); (B) The amplification results of inoculated leaf with *NtEF1α* specific primers (F: 5′- TGAGATGCACCACGAAGCTC-3′; R: 5′- CCAACATTGTCACCAGGAAGTG-3′); (C) The amplification results of upmost fully expanded leaf with CMV *coat protein* gene specific primers; (D) The amplification results of upmost fully expanded leaf with *NtEF1α* specific primers. M, DNA molecular weight marker; 1–4, mock-inoculated, CMV-inoculated at 1 dpi, CMV-inoculated at 3 dpi, and CMV-inoculated at 5 dpi of NC82 leaf; 5–8, mock-inoculated, CMV-inoculated at 1 dpi, CMV-inoculated at 3 dpi, and CMV-inoculated at 5 dpi of Taiyan8 leaf.

**Supplementary Figure 3**. Unigene annotation success rates across multiple databases.

**Supplementary Table 1**. Length distribution of assembled transcripts and unigenes.

**Supplementary Table 2**. Summary of the clean reads mapping to the Trinity-assembled transcriptome.

**Supplementary Table 3**. Annotation of differentially expressed genes in two tobacco cultivars at different time points (NB for NC82, TB for Taiyan8).

**Supplementary Table 4**. Expression (FPKM) of DEGs in two tobacco cultivars at different time points (NB for NC82, TB for Taiyan8).

**Supplementary Table 5**. Expression and annotation of 20 common differentially expressed genes in two tobacco cultivars at different time points (NB for NC82, TB for Taiyan8).

**Supplementary Table 6**. Gene ontology (GO) enrichment of differentially expressed genes in NC82 and Taiyan8.

**Supplementary Table 7**. Numbers of differentially expressed genes among the top 20 metabolism pathways in NC82 and Taiyan8, determined by Kyoto Encyclopedia of Genes and Genomes (KEGG) enrichment analysis.

**Supplementary Table 8**. Differentially expressed genes involved in photosynthesis, reactive oxygen species (ROS) scavenging, signal transduction, and plant–pathogen interaction in NC82 and Taiyan8.

**Supplementary Table 9**. Specific primers of differential gene sequences for real-time quantitative reverse transcription polymerase chain reaction (qRT-PCR).


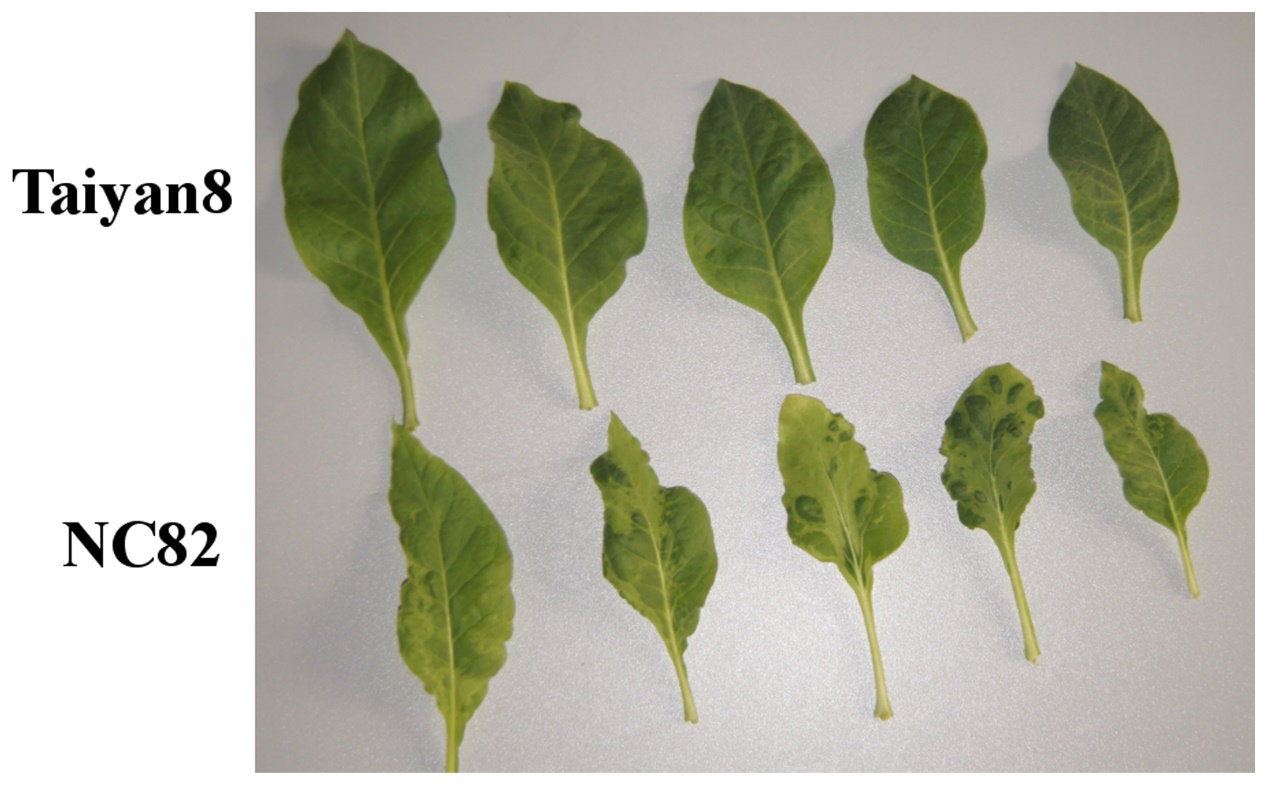


Supplementary Figure 1. Symptoms of *Cucumber mosaic virus* (CMV) infection on systemic leaves of NC82and Taiyan8 at 30 days post inoculation (dpi). NC82 showed severe chlorosis and leaf distortion; Taiyan8 showed slighter chlorosis and leaf vein clearing.


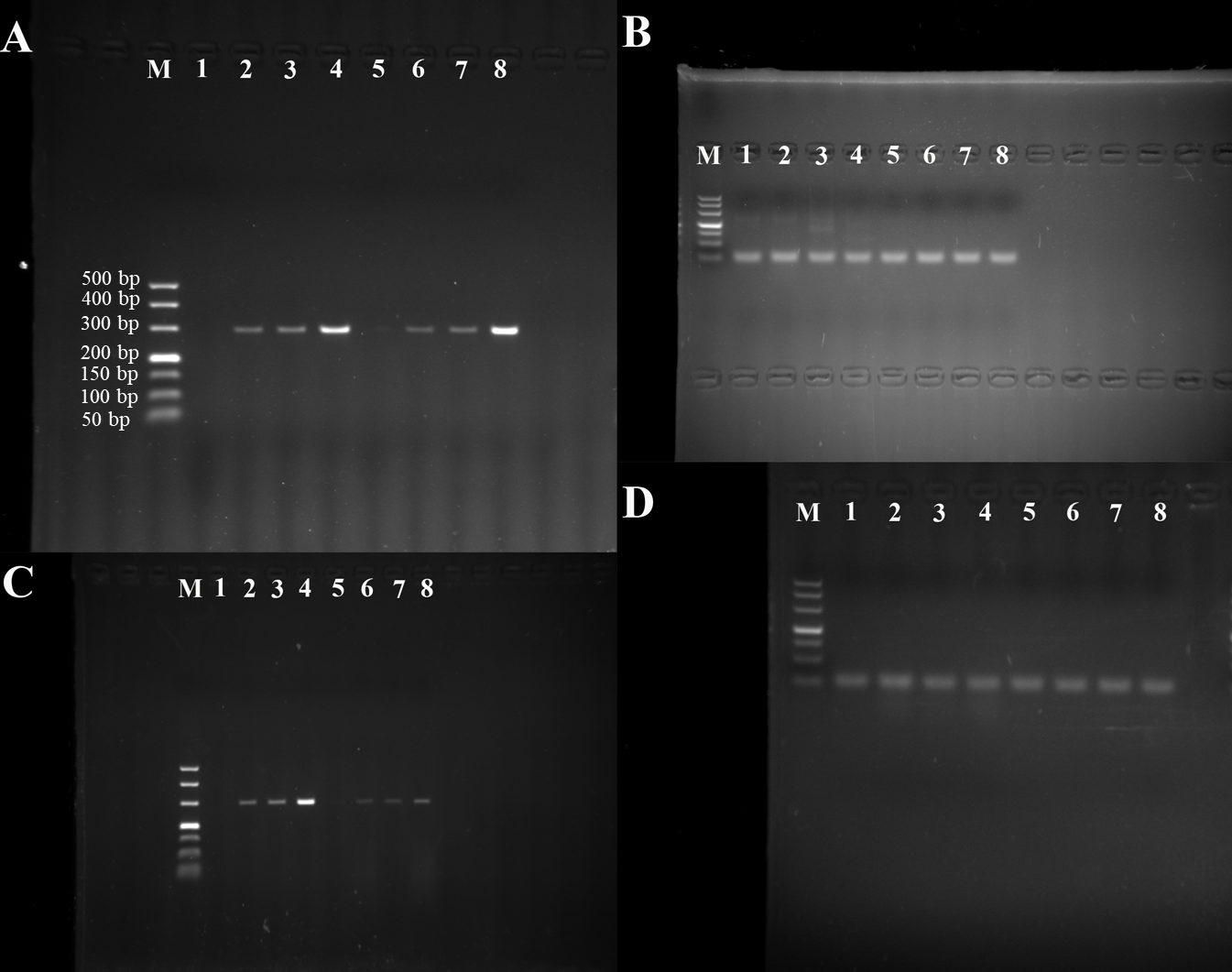


Supplementary Figure 2. The full-length agarose gel electrophoresis of semiquantitative RT-PCR detection results of inoculated and upmost fully expanded leaves. (A) The amplification results of inoculated leaf with CMV *coat protein* gene specific primers (F: 5′-TACCCTGAAACCACCGAAAA-3′; R: 5′-CGCCGAAAGATCATACAACA-3′); (B) The amplification results of inoculated leaf with *NtEF1α* specific primers (F: 5′- TGAGATGCACCACGAAGCTC-3′; R: 5′- CCAACATTGTCACCAGGAAGTG-3′); (C) The amplification results of upmost fully expanded leaf with CMV *coat protein* gene specific primers; (D) The amplification results of upmost fully expanded leaf with *NtEF1α* specific primers. M, DNA molecular weight marker (DL500 DNA Marker, Takara, Beijing, China); 1–4, mock-inoculated, CMV-inoculated at 1 dpi, CMV-inoculated at 3 dpi, and CMV-inoculated at 5 dpi of NC82 leaf; 5–8, mock-inoculated, CMV-inoculated at 1 dpi, CMV-inoculated at 3 dpi, and CMV-inoculated at 5 dpi of Taiyan8 leaf.


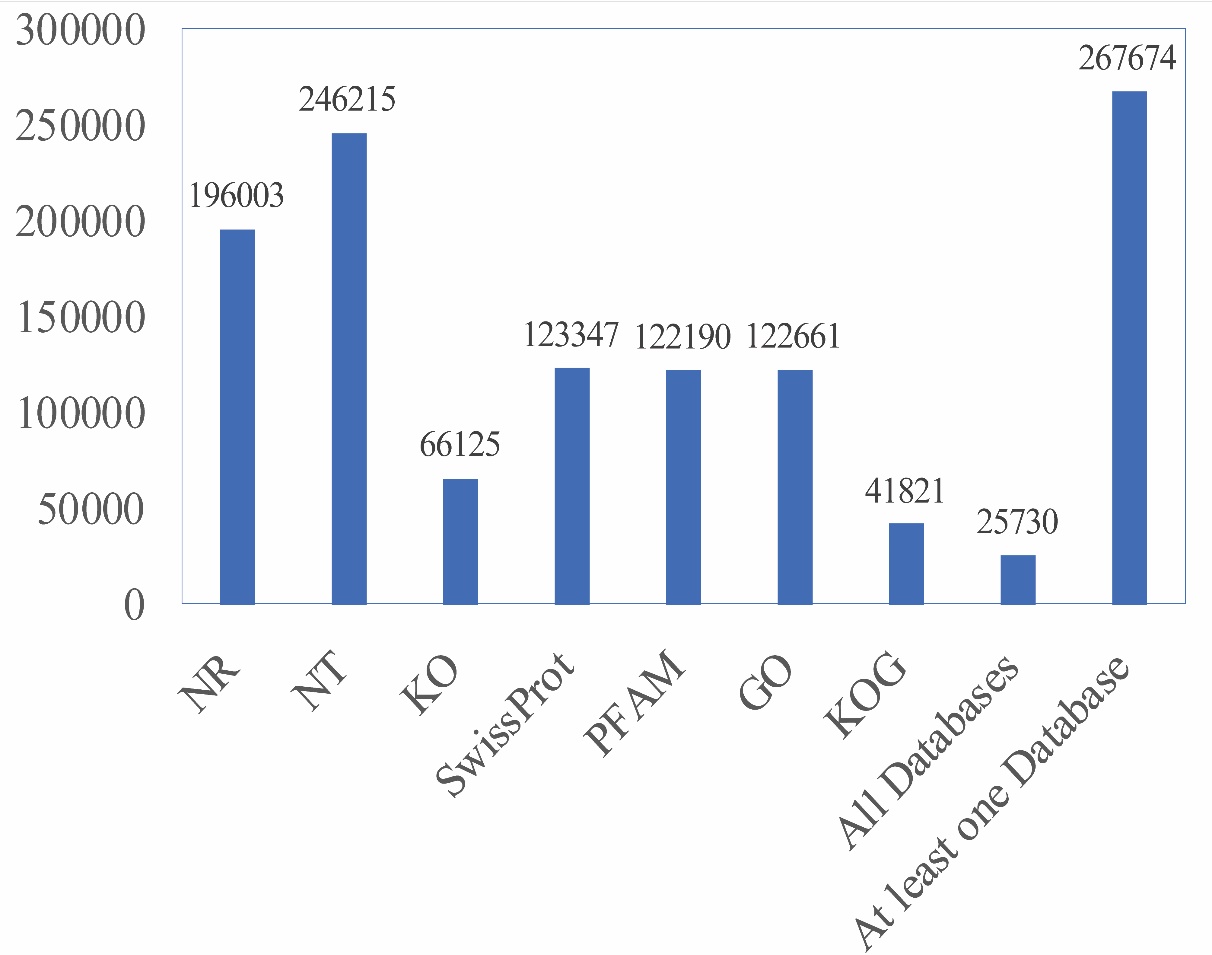


Supplementary Figure 3. Unigene annotation success rates across multiple databases.
